# Fruitflow inhibits platelet function by suppressing Akt/GSK3β, Syk/PLCγ2 and p38 MAPK phosphorylation in collagen-stimulated platelets

**DOI:** 10.1186/s12906-022-03558-5

**Published:** 2022-03-17

**Authors:** Huilian Chen, Shenghao Zhang, Hui Wang, Li Bao, Wei Wu, Ruomei Qi

**Affiliations:** 1 The Key Laboratory of Geriatrics, Beijing Institute of Geriatrics, Institute of Geriatric Medicine, Chinese Academy of Medical Sciences, Beijing Hospital/National Center of Gerontology of National Health Commission, Beijing, P. R. China; 2 Graduate School of Peking Union Medical College, Chinese Academy of Medical Sciences, Beijing, P. R. China

**Keywords:** Fruitflow, Platelets, TXB_2_, PF4, Akt, GSK3β, Syk, PLCγ2

## Abstract

**Background:**

Platelets play an important role in the progression of atherosclerosis and cardiovascular events. The inhibition of platelet function is a main strategy to reduce risk of cardiovascular events. Some studies have shown that tomato extracts inhibit platelet function, but the molecular mechanisms remain unclear. Fruitflow is a water-solute tomato extract and the main ingredients including flavonoids, adenosine, chlorogenic acid, phytosterols, naringenin, and carotenoids. The present study investigated the effects of fruitflow on adenosine diphosphate (ADP)- and collagen- stimulated platelet aggregation, platelet adhesion, and levels of thromboxane B_2_ (TXB_2_), 6-keto-prostaglandin F_1α_ (PGF_1α_), and platelet factor 4 (PF4) and explored the underlying molecular mechanisms.

**Methods:**

Platelet-rich plasma (PRP) was used for measurement of platelet aggregation, TXB_2_, 6-keto- PGF_1α_, and PF4 levels. Platelet aggregation was analyzed using a Chrono-Log aggregometer. TXB_2_, 6-keto- PGF_1α_, and PF4 levels were determined using enzyme-linked immunosorbent assay kits. Immunoblotting was used to detect protein expression and phosphorylation on washed platelets. Platelet adhesion and spreading were determined by immunofluorescence.

**Results:**

Fruitflow (1, 3, 10 and 100 μg/ml) dose-dependently inhibited platelet aggregation that was induced by ADP and collagen. Fruitflow (100 μg/ml) treatment completely suppressed ADP- and collagen-stimulated platelet aggregation. Fruitflow (100 μg/ml) significantly decreased TXB_2_ and 6-keto-PGF_1α_ generation and PF4 release in ADP- and collagen-stimulated platelets. Treatment with fruitflow effectively blocked collagen-induced platelet spreading. To determine the potential molecule mechanism of action of fruitflow, we investigated the protein expression and phosphorylation of several signaling molecules in collagen-activated platelets. Fruitflow dose-dependently suppressed Akt, Glycogen synthase kinase-3β (GSK-3β), spleen tyrosine kinase (Syk) and phospholipase Cγ2 (PLCγ2) and p38 MAPK phosphorylation that was induced by collagen.

**Conclusion:**

Fruitflow inhibited platelet aggregation and reduced TXB_2_, 6-keto-PGF1_α_, and PF4 levels in ADP- and collagen-stimulated platelets. The mechanism of action of fruitflow may be associated with the suppression of Akt/GSK3β, Syk/PLCγ2, and p38 MAPK phosphorylation in collagen-activated platelets. Fruitflow is a natural product derived from tomato and can be used as a health food for decreasing platelet activity.

**Supplementary Information:**

The online version contains supplementary material available at 10.1186/s12906-022-03558-5.

## Background

Cardiovascular disease has the highest mortality rate worldwide [1, 2]. The direct cause of death from cardiovascular events is coronary thrombosis. Atherosclerosis is considered an inflammatory disease of systemic arteries [3, 4]. Platelets participate in thrombosis and the early inflammatory progression of atherosclerosis [5–7]. Platelets contain numerous α-granules, dense granules, and lysosomes. Upon platelet activation, a series of responses occurs, including changes in shape, aggregation, and the migration of granules to the cell surface that release various factors, such as platelet factor 4 (PF4), CD40 ligand, and adenosine diphosphate (ADP), etc. [8, 9]. The phosphoinositide 3-kinase (PI3K)/Akt signaling pathway is involved in platelet activation that is induced by multiple stimulants. Akt is a serine/threonine kinase. Its phosphorylation plays an important role in promoting granule secretion and platelet aggregation [10, 11]. Platelet spreading is an early consequence of integrin-mediated outside-in signaling and represents outward movement of the cell membrane, characterized by the formation of lamellipodia and filipodia [12, 13].

Glycogen synthase kinase (GSK) is a widely expressed cytoplasmic serine/threonine protein kinase. It exists as two high homologous isoforms, GSK3α and GSK3β, that are regulated in a similar manner [14]. Previous studies have reported different functions of GSK3α and GSK3β [15, 16]. GSK is a downstream molecule of Akt [17]. However, the role of GSK in platelet activation is still not fully understood.

Tyrosine kinase Syk play a critical role on collagen- and thrombin-induced platelet activation [18, 19]. Phospholipase Cγ2 (PLCγ2) is an important signaling molecule in the intracellular signaling cascade mediated by collagen receptor GPVI, whose activation leads to the production of second messenger inositol triphosphate (IP3) and diacylglycerol (DG), followed by increased intracellular calcium concentration [20]. Both Syk and PLCγ2 are rapidly phosphorylated in collagen-stimulated platelets [21].

Epidemiological studies have shown that the Mediterranean diet can effectively reduce the risk of cardiovascular disease. The Mediterranean diet mainly consists of tomatoes, green vegetables, fresh fish, olive oil, and red wine. However, the ways in which this diet affects cell function remains unclear. Previous studies have shown that some components of tomatoes affect platelet function [22–24]. Fruitflow (FF) is a commercially available water-soluble tomato extract, the main ingredients include flavonoids, adenosine, chlorogenic acid, phytosterols, naringenin, and carotenoids [22]. This water-soluble tomato extract has been shown to inhibit platelet function and angiotensin-converting enzyme and relax the vascular endothelium. O’Kennedy et al. recently reported that FF significantly reduced agonist-stimulated platelet aggregation in healthy subjects [25]. However, the effects of FF on platelet function awaits further in vitro investigation. The present study evaluated the effects of FF on ADP- and collagen-induced platelet aggregation, platelet spreading, and the levels of platelet-releasing factors and investigated the potential mechanism of action.

## Methods

### Materials

Fruitflow was provided by By-Health Co., Ltd. (Zhuhai, Guangdong, China). Aspirin (acetylsalicylic acid) was purchased from Sigma-Aldrich (St. Louis, MO, USA). Collagen and ADP were purchased from Chrono-Log (Havertown, PA, USA). Human platelet factor 4 (PF4; CXCL4), thromboxane B_2_ (TXB_2_), and 6-keto-prostaglandin F_1α_ (PGF_1α_) enzyme-linked immunosorbent assay (ELISA) kits were purchased from Abcam (Boston, MA, USA). Polyclonal anti-Akt antibody and monoclonal anti-Syk antibody were purchased from Santa Cruz Biotechnology (Santa Cruz, CA, USA). Monoclonal anti-phospho-Akt (Ser473) antibody, monoclonal anti-GSK3β antibody, monoclonal anti-phospho-GSK3β (Ser9) antibody, polyclonal anti-p38 mitogen-activated protein kinase (MAPK) antibody, polyclonal anti-phospho-p38 MAPK (Thr180/Tyr182) antibody, monoclonal anti-phospho-PLCγ2 (Tyr759) antibody, and polyclonal anti- PLCγ2 antibody were purchased from Cell Signaling Technology (Danvers, MA, USA). Monoclonal anti-phospho-syk (Tyr525) were purchased from Abcam (Boston, MA, USA). Phalloidin-iFluor 555 was purchased from Abcam (Boston, MA, USA), which is one of a series of phalloidin conjugates that bind to actin filaments.

### Analysis of platelet aggregation

The experiments were conducted according to the principles of the Declaration of Helsinki (World Medical Association, 2013). The healthy donors, aged up to 45 years old, no gender restrictions, and had not taken any medication for 2 weeks. The donors provided written informed consent to confirm the blood sample was used only in this study.

Blood samples were collected into 3.2% sodium citrate vacuum anticoagulation tubes. The blood samples were centrifuged at 200×*g* for 15 min to obtain platelet-rich plasma (PRP). The PRP (300 μl) was preincubated with various doses of FF (1, 3, 10, 30, and 100 μg/ml) or aspirin (10, 30, 100, and 300 μM) for 5 min, and then ADP (5 μM) or collagen (5 μg/ml) was added to induce platelet aggregation. Platelet aggregation was measured using a Chrono-Log aggregometer (Chrono-Log, Havertown, PA, USA).

### Measurement of TXB_2_, 6-keto-PGF_1α_, and PF4 levels by ELISA

Blood was collected into 3.2% sodium citrate vacuum anticoagulation tubes from healthy volunteers who had not taken any medication for at least 2 weeks before the study. The blood samples were centrifuged at 200×*g* to obtain PRP. The PRP (300 μl) was preincubated with various doses of fruitflow or aspirin for 5 min, and then the platelet agonist ADP (5 μM) or collagen (5 μg/ml) was added for another 5 min. The reaction was stopped by the addition of 2 mM ethylenediaminetetraacetic acid (EDTA). The levels of TXB_2_, 6-keto-PGF_1α_, and PF4 were determined using ELISA kits (Abcam, Boston, MA, USA) according to the manufacturer’s instructions.

### Platelet spreading on immobilized fibrinogen

Platelet-rich plasma was obtained from whole blood by centrifugation at 200×*g* for 10 min. The PRP was then centrifuged at 200×*g* for 10 min in the presence of ACD and 2 mM ethylenediaminetetraacetic acid (EDTA), washed twice with modified Tyrode’s buffer (138 mM NaCl, 3.3 mM NaH_2_PO_4_▪2H_2_O, 1 mM MgCl_2_, 2.9 mM KCl, 5.5 mM glucose, and 20 mM HEPES), and resuspended in modified Tyrode’s buffer. Glass slides were coated with 20 μg/ml fibrinogen overnight, and then a 2 × 10^6^ washed platelet suspension (200 μl) was added on the glass slides for 1 h at room temperature to allow platelet adherence and spread on fibrinogen-coated wells. Non-adherent platelets were removed by aspiration, washed twice with phosphate-buffered saline (PBS), fixed with 4% paraformaldehyde, permeabilized by the addition of 0.1% TritonX-100, and stained with Phalloidin-iFluor 555 for 1 h. Platelet spreading was visualized by fluorescence microscopy.

### Western blot assay

Washed platelets (1 × 10^9^/ml) were preincubated with fruitflow (1, 10, and 100 μg/ml) for 5 min, and then collagen was added to the cuvette for 5 min to determine Akt, GSK3β and p38 MAPK phosphorylation, or 30 s to determine Syk and PLCγ2 phosphorylation. Whole cell lysates were separated by sodium dodecyl sulfate-polyacrylamide gel electrophoresis (PAGE) and transferred to polyvinylidene membranes. The membranes were incubated overnight with specific primary antibodies (1:1000 dilution) at 4 °C and then incubated with anti-mouse or anti-rabbit antibodies (1:5000 dilution). The bands were exposed using electrochemiluminescent reagent and the EvolutionCapt system (Vilber Lourmat) and quantified using ImagePro Plus software.

### Statistical analysis

Quantitative data are presented as the mean ± SEM. Significant differences between two groups were analyzed using two-tail unpaired Student’s *t*-test. Statistical significance among multiple groups was analyzed using one-way analysis of variance (ANOVA) followed by the Student-Newman-Keuls post hoc test. All of the analyses were performed using SPSS 25.0 software (Armonk, NY, USA). Values of *p* < 0.05 were considered statistically significant.

## Results

### Fruitflow inhibited ADP- and collagen-induced platelet aggregation

**Fig. 1:**
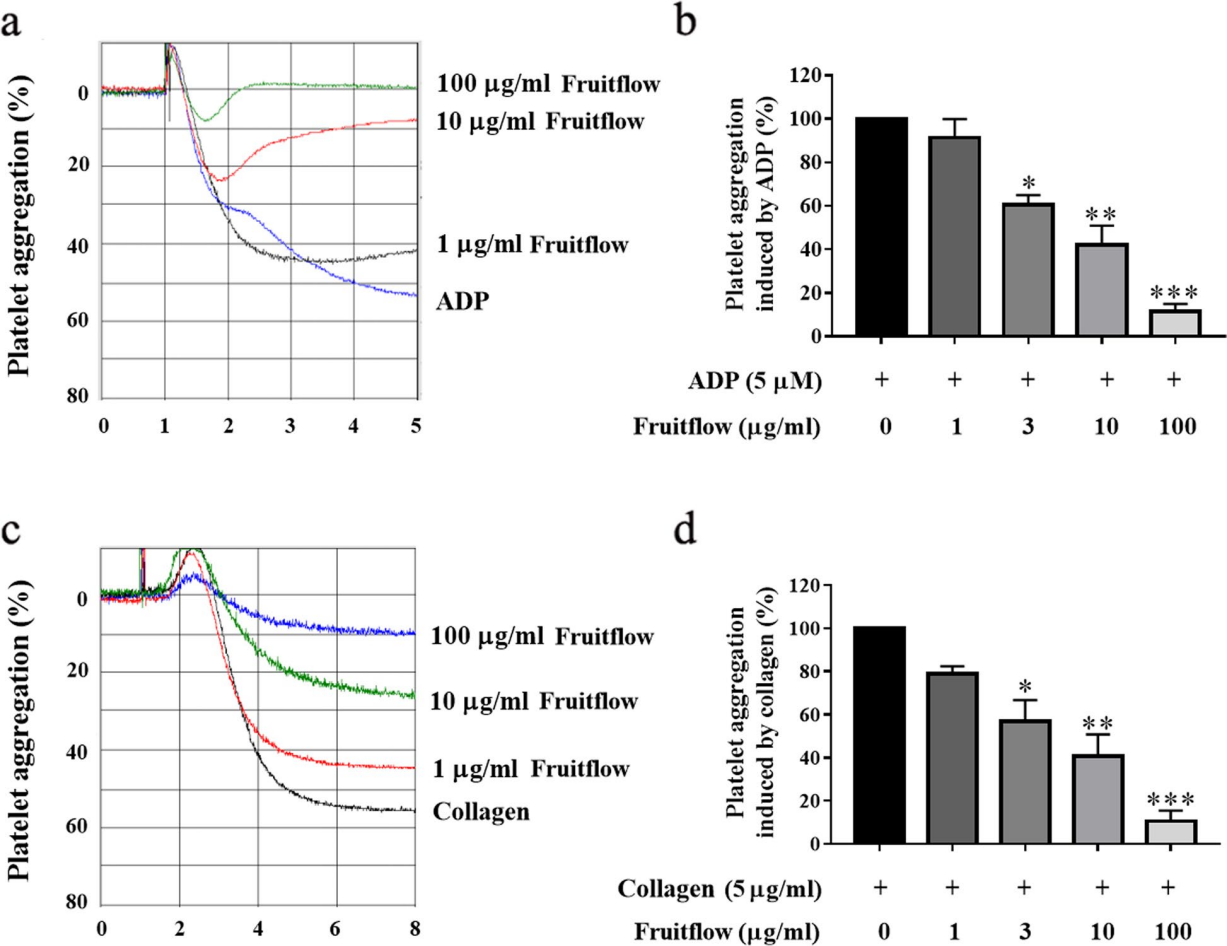
Fruitflow inhibited ADP- and collagen-induced platelet aggregation. Platelet-rich plasma was treated with various doses of FF for 5 min, and then the cuvette was set in the platelet aggregation assay channel with stirring for 1 min. Adenosine diphosphate or collagen was then added to induce platelet aggregation. **a** Fruitflow dose-dependently inhibited platelet aggregation that was induced by ADP. **b** Analysis of platelet aggregation in ADP-stimulated platelets. **c** Fruitflow dose-dependently inhibited platelet aggregation that was induced by collagen. **d** Analysis of platelet aggregation in collagen-stimulated platelets. The data were obtained from five independent experiments. *p < 0.05, **p < 0.01, ***p < 0.001, significant difference between non-FF-treated and FF-treated platelets

We first determined the effect of fruitflow on platelet aggregation in vitro. Platelet-rich plasma was preincubated with various doses of fruitflow (1, 3, 10 and 100 μg/ml) for 5 min, and then ADP or collagen was added to induce platelet aggregation. As shown in Fig. 1, fruitflow dose-dependently inhibited platelet aggregation that was induced by ADP, and 100 μg/ml fruitflow almost completely inhibited ADP-induced aggregation. Fruitflow also dose-dependently suppressed platelet aggregation that was induced by collagen, and 100 μg/ml fruitflow potently inhibited collagen-stimulated platelet aggregation.

### Fruitflow and aspirin synergistically inhibited ADP- and collagen-induced platelet aggregation

**Fig. 2:**
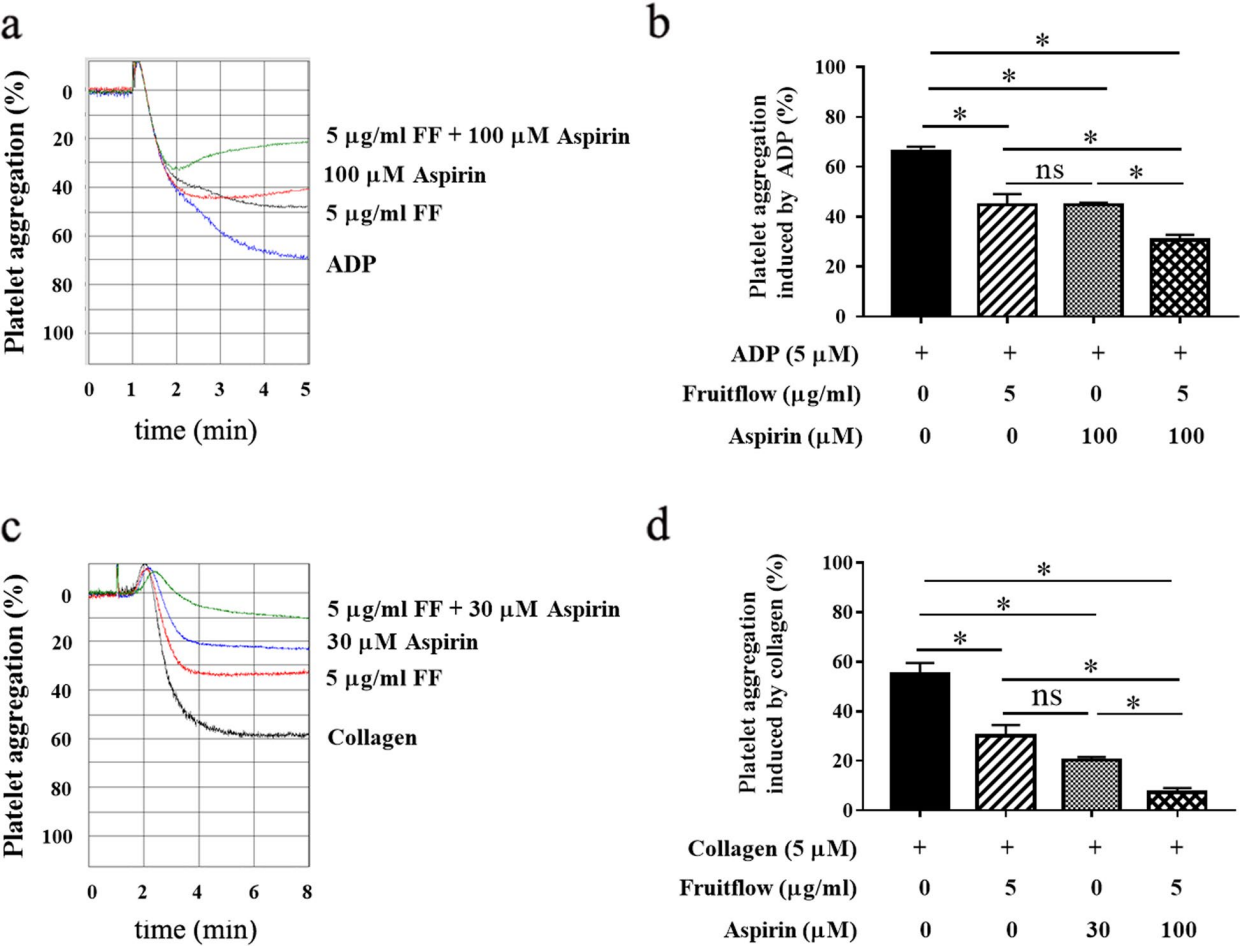
Fruitflow and aspirin synergistically inhibited platelet aggregation that was induced by ADP and collagen. **a, b** The combination of low-dose FF and aspirin inhibited platelet aggregation that was induced by ADP. **c, d** The combination of low-dose FF and aspirin inhibited platelet aggregation that was induced by collagen. The data were obtained from five independent experiments. *p < 0.05, significant difference between groups

To determine whether fruitflow and aspirin exert synergistic inhibitory effects on platelet aggregation, we first determined the effects of aspirin on ADP- and collagen-stimulated platelet aggregation. Our preliminary experiments showed that aspirin had a better inhibitory effect on collagen-stimulated platelets, and 30 μM aspirin inhibited platelet aggregation by approximately 60%. Aspirin had a weaker inhibitory effect on ADP-induced platelet aggregation, and the higher concentration of 100 μM was needed to inhibit platelet aggregation by nearly 50%. Therefore, we used a combination of 5 μg/ml fruitflow and 100 μM aspirin to evaluate their possible synergistic inhibitory effects on ADP-induced platelet aggregation. For collagen-induced platelet aggregation, we used 5 μg/ml fruitflow and 30 μM aspirin. Platelet aggregation was first analyzed in ADP-stimulated platelets. As shown in Fig. 2A and B, 5 μg/ml fruitflow decreased the rate of ADP-induced platelet aggregation by 36.1%, and 100 μM aspirin decreased the rate of ADP-induced platelet aggregation by 37.3%. The combination of 5 μg/ml fruitflow and 100 μM aspirin decreased the rate of ADP-induced platelet aggregation by 54.5%. As shown in Fig. 2C and D, 5 μg/ml fruitflow decreased the rate of collagen-induced platelet aggregation by 45.5%, and 30 μM aspirin decreased the rate of collagen-induced platelet aggregation by 63.1%. The combination of 5 μg/ml fruitflow and 100 μM aspirin decreased the rate of collagen-induced platelet aggregation by 86.7%. The effects of the combination of fruitflow and aspirin were significantly different from either treatment alone.

### Fruitflow reduced TXB_2_ and 6-keto-PGF_1α_ levels in ADP- and collagen-activated platelets

**Fig. 3:**
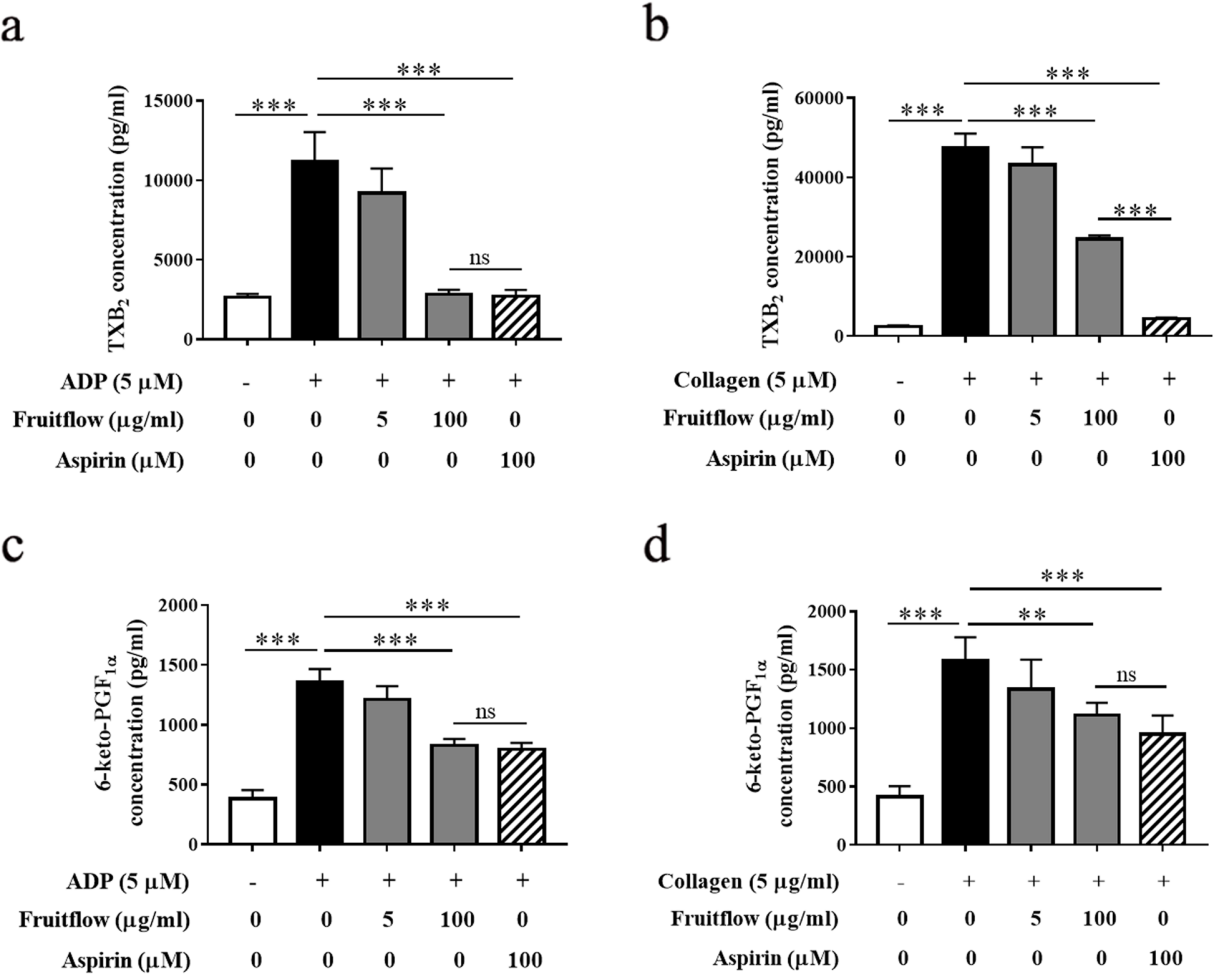
Fruitflow decreased TXB_2_ and 6-keto-PGF_1α_ levels in ADP- and collagen-activated platelets. Platelet-rich plasma was treated with various doses of FF and aspirin for 5 min. Adenosine diphosphate or collagen was then added to induce platelet activation. The levels of TXB_2_ and 6-keto**-**PGF_1α_ were measured using ELISA kits. **a** Fruitflow and aspirin reduced TXB_2_ generation that was induced by ADP. **b** Fruitflow and aspirin reduced TXB_2_ generation that was induced by collagen. **c** Fruitflow and aspirin reduced 6-keto**-**PGF_1α_ production that was induced by ADP. **d** Fruitflow and aspirin reduced 6-keto**-**PGF_1α_ production that was induced by collagen. The data were obtained from five independent experiments. *p < 0.05, **p < 0.01, significant difference between groups

To examine whether fruitflow affects arachidonic acid metabolism, we analyzed the levels of TXB_2_ and PGF1α in ADP- and collagen-activated platelets. The content of TXB_2_ and 6-keto-PGF_1α_ was detected using ELISA kits. As shown in Fig. 3A and B, TXB_2_ levels increased 4.2-fold in ADP-activated platelets. Fruitflow (100 μg/ml) and aspirin (100 μM) completely abolished ADP-induced TXB_2_ generation. TXB_2_ levels increased 19.5-fold in collagen-activated platelets. Treatment with 100 μg/ml fruitflow partially reduced TXB_2_ levels by 48.7% in collagen-stimulated platelets, whereas treatment with 100 μM aspirin completely abolished collagen-induced TXB_2_ generation. As shown in Fig. 3C and D, 6-keto-PGF_1α_ levels increased 3.5-fold in ADP-activated platelets. Fruitflow (100 μg/ml) significantly decreased 6-keto-PGF_1α_ generation by 39.0%. Treatment with aspirin (100 μM) decreased 6-keto-PGF1α levels by 41.4% in ADP-activated platelets. The levels of 6-keto-PGF_1α_ increased 4.3-fold in collagen-activated platelets. Fruitflow (100 μg/ml) decreased 6-keto-PGF_1α_ levels by 37.4%, and aspirin (100 μM) decreased 6-keto-PGF_1α_ levels by 46.3% in collagen-activated platelets. These results indicated that aspirin had a better inhibitory effect on TXB_2_ generation than FF in collagen-activated platelets.

### Fruitflow decreased PF4 levels in ADP- and collagen-activated platelets

**Fig. 4:**
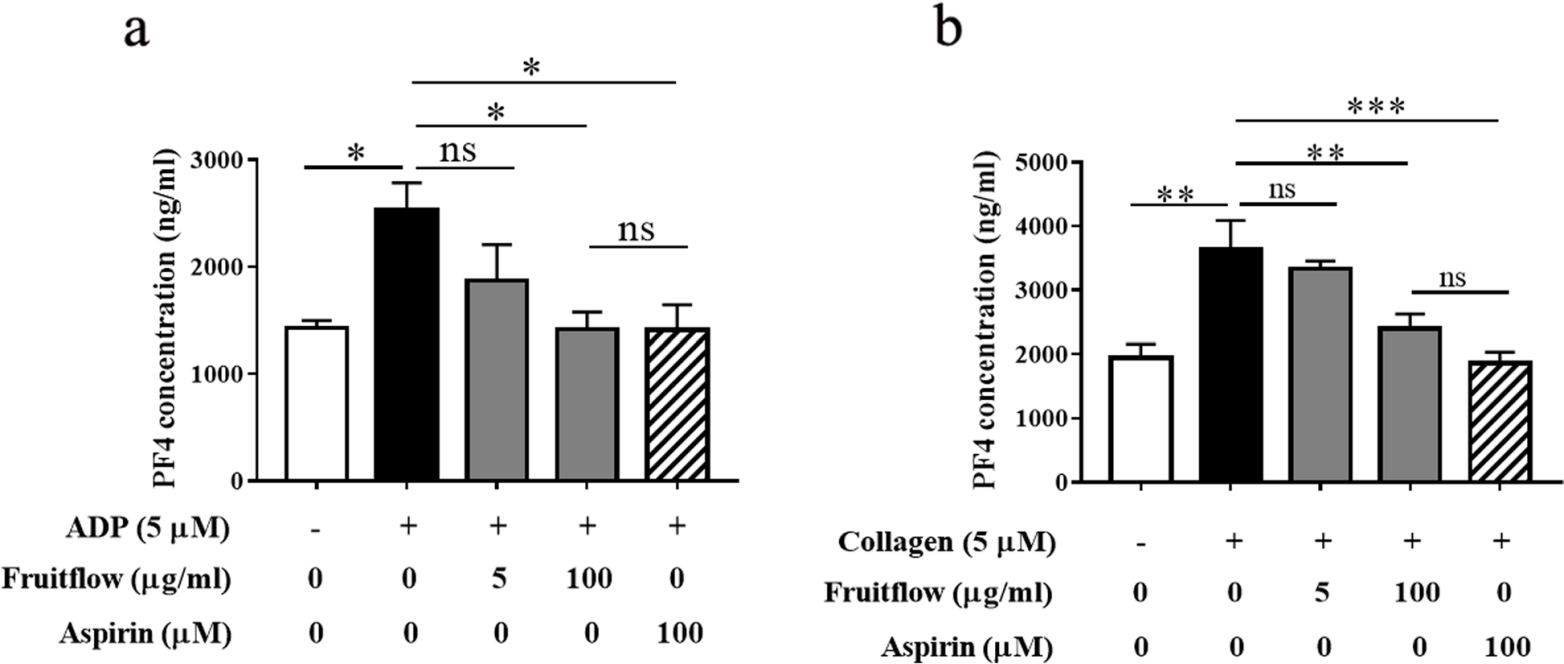
Fruitflow decreased PF4 levels in ADP- and collagen-activated platelets. Platelet-rich plasma was treated with various doses of FF and aspirin for 5 min. Adenosine diphosphate or collagen was then added to induce platelet activation. The content of PF4 was measured using an ELISA kit. **a** Fruitflow and aspirin decreased PF4 production that was induced by ADP. **b** Fruitflow and aspirin decreased PF4 production that was induced by collagen. The data were obtained from five independent experiments. *p < 0.05, significant difference between groups

Platelet factor 4 is an inflammatory mediator that is stored in α-granules of platelets. Platelet factor 4 has been shown to be involved in various inflammatory responses, including vascular inflammation and atherosclerosis. Therefore, we examined the effects of fruitflow on PF4 levels in ADP- and collagen-activated platelets. As shown in Fig. 4A and B, ADP stimulation increased PF4 levels 1.8-fold, and fruitflow (100 μg/ml) completely suppressed the ADP-induced increase in PF4 levels. Treatment with aspirin (100 μM) exerted a similar effect. Collagen stimulation increased PF4 levels 2.3-fold in activated platelets, and fruitflow (100 μg/ml) completely suppressed the collagen-induced increase in PF4 levels. Treatment with aspirin (100 μM) exerted similar effects.

### Fruitflow inhibited platelet spreading

**Fig. 5:**
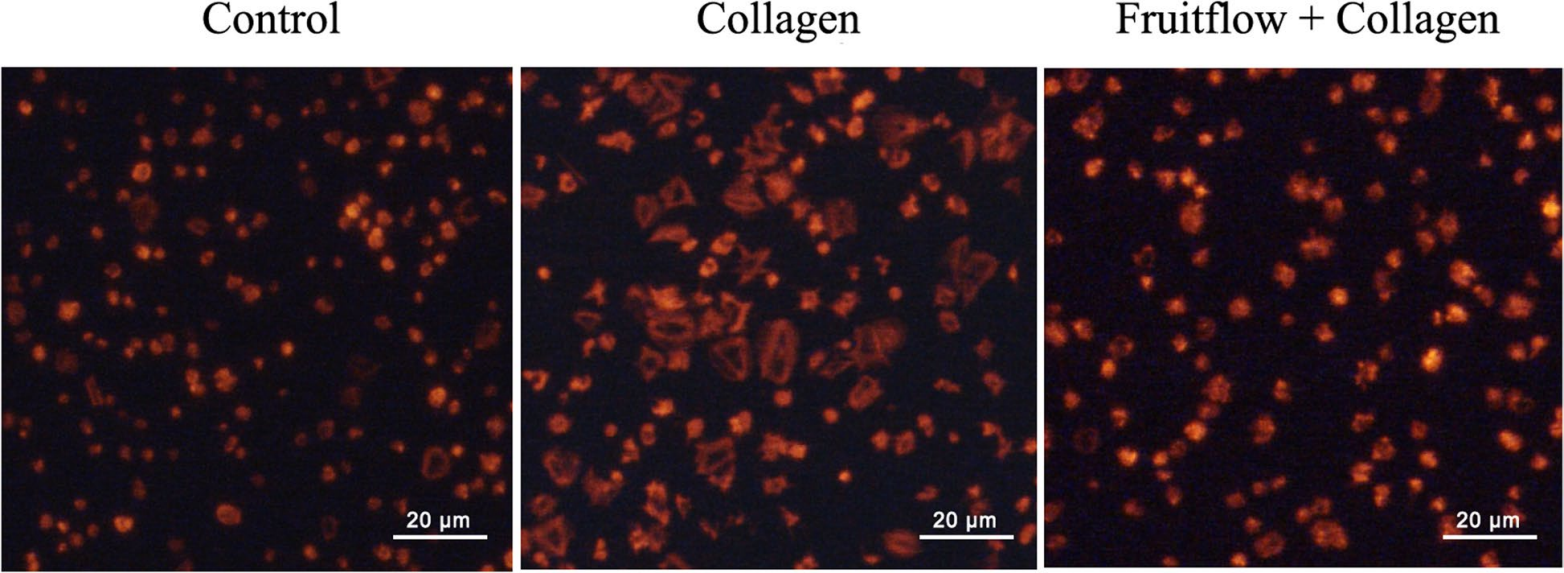
Fruitflow inhibited platelet spreading on immobilized fibrinogen. Washed platelets were incubated with FF (100 μg/ml) for 5 min, and then collagen (1 μg/ml) was added for 10 min. Cells were then placed on fibrinogen-covered slide wells for 1 h, stained with Phalloidin-iFluor 555 for 1 h, and observed under a fluorescence microscope. The control group was treated with PBS. The data were obtained from five independent experiments

Platelet spreading is an important feature of morphological changes whereby platelets adhere to damaged vascular endothelial cells and subcellular matrix components. To determine whether fruitflow affects platelet spreading, we observed platelet spreading using immunofluorescence. Washed platelets were treated with fruitflow (100 μg/ml) for 5 min, and then collagen was added for another 10 min. The platelets were then placed on a fibrinogen-coated well for 1 h. As shown in Fig. 5, in the untreated control group, platelets adhered to the fibrinogen-coated well but exhibited less spreading. The treatment of platelets with collagen (1 μg/ml) significantly induced platelet spreading, and fruitflow significantly abolished collagen-stimulated platelet spreading.

### Fruitflow suppressed Akt, GSK3β, Syk, PLCγ2 and p38MAPK phosphorylation in collagen-stimulated platelets

**Fig. 6:**
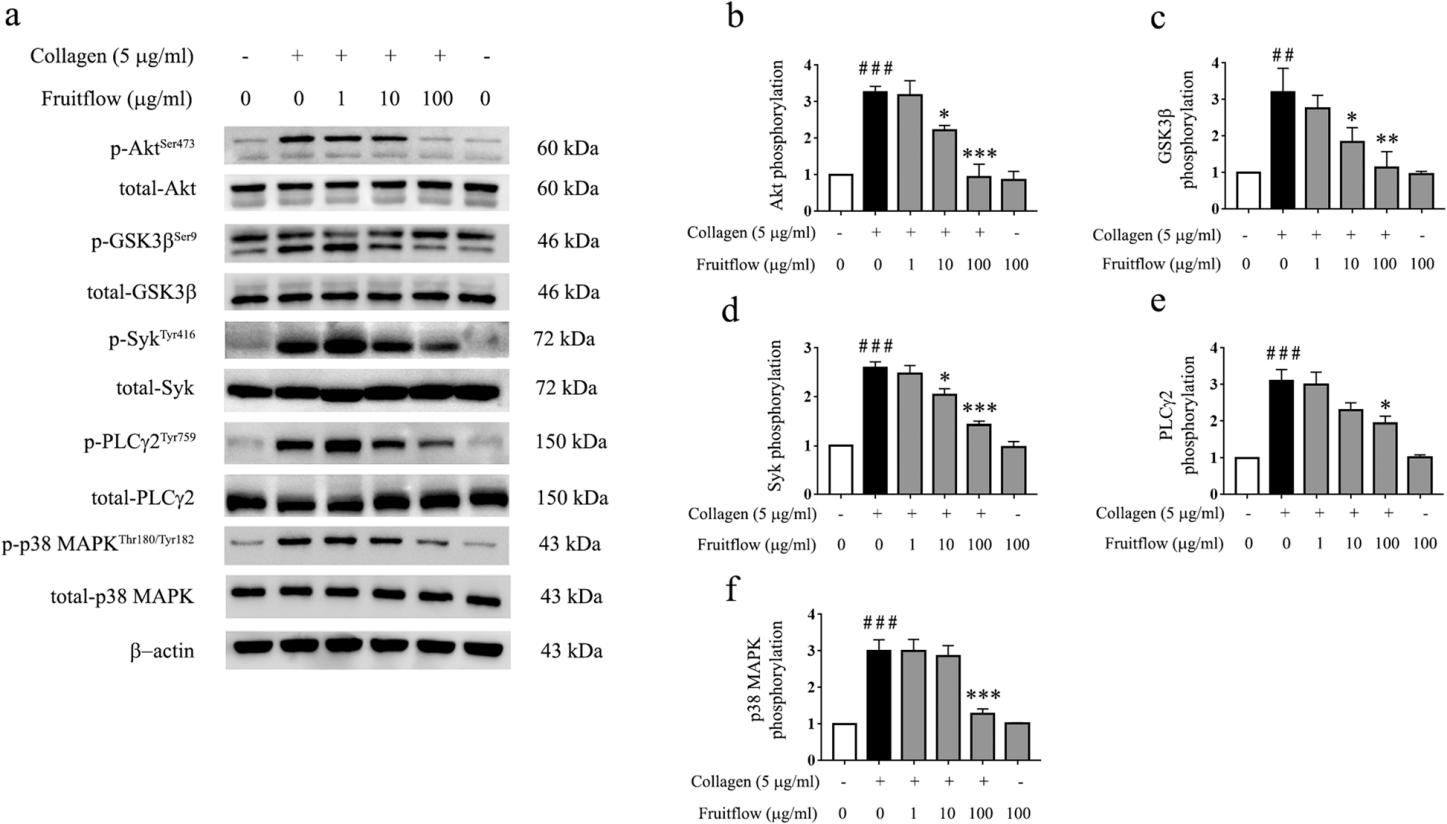
Fruitflow suppressed Akt, GSK3β, Syk, PLCγ2 and p38 MAPK phosphorylation in collagen-stimulated platelets. **a** Fruitflow inhibited Akt, GSK3β, Syk, PLCγ2 and p38 MAPK phosphorylation in collagen-stimulated platelets. **b** Density analysis of Akt phosphorylation. **c** Density analysis of GSK3β phosphorylation. **d** Density analysis of Syk phosphorylation. **e** Density analysis of PLCγ2 phosphorylation. **f** Density analysis of p38 MAPK phosphorylation. The data were obtained from three independent experiments. ^##^*p* < 0.01, ^###^*p* < 0.001, significant difference between control and collagen-treated platelets. *p < 0.05, **p < 0.01, ***p < 0.001, significant difference between FF-treated platelets and untreated platelets in collagen-activated platelets. (In order to improve the clarity and conciseness of the presentation of the western blot results, we cropped the original figures. And the original uncropped figures are shown in supplementary material)

In order to investigate the molecule mechanisms of action of fruitflow inhibiting platelet activation, we determined Akt, GSK3β, Syk, PLCγ2 and p38 MAPK phosphorylation in collagen-stimulated platelets. As shown in Fig. 6, collagen stimulation increased the levels of Akt, GSK3β, Syk, PLCγ2 and p38 MAPK phosphorylation, and fruitflow treatment (100 μg/ml) completely abolished their phosphorylation that was induced by collagen. The results suggest that the inhibitory effect of fruitflow on platelet activation might be associated with the suppression of Akt, GSK3β, Syk, PLCγ2, and p38 MAPK phosphorylation.

## Discussion

In the present study, we investigated the effect of the water-soluble tomato extract fruitflow on platelet function. Our results indicated that fruitflow inhibited platelet aggregation that was induced by ADP and collagen and enhanced the inhibitory effect of aspirin on platelet aggregation. Moreover, fruitflow decreased the levels of TXB_2_ and 6-keto-PGF_1α_ in ADP- and collagen-activated platelets. Both TXB_2_ and 6-keto-PGF_1α_ are metabolites of arachidonic acid. Previous studies demonstrated that TXB_2_ levels are higher and 6-keto-PGF_1α_ levels are lower in several diseases, such as cardiovascular disease and chronic pulmonary heart disease [26, 27]. Our results showed that 100 μg/ml fruitflow and 100 μM aspirin similarly attenuated TXB_2_ generation in ADP-activated platelets. However, 100 μg/ml fruitflow only partially inhibited TXB_2_ generation in collagen-activated platelets, whereas 100 μM aspirin completely inhibited TXB_2_ generation. In addition, a previous study reported that tomato extract (20–50 μl of 100% juice) inhibited both ADP- and collagen-induced platelet aggregation by 70% but could not inhibit arachidonic acid-induced platelet aggregation and concomitant thromboxane synthesis under similar experiment condition [28]. These findings show that the mechanism of action of fruitflow is different from aspirin in inhibiting TXB_2_. Aspirin is an irreversible inhibitor of cyclooxygenase-1 (COX-1), whereas the effect of FF on COX-1 may be indirect [22, 29, 30]. Our results showed that neither fruitflow nor aspirin completely prevented the production of 6-keto-PGF_1α_ in ADP- and collagen-activated platelets.

Platelet factor 4, also called CXCL4, belongs to the chemokine family [31]. It is stored in α-granules of platelets and is the most abundant protein in α-granules. Platelet factor 4 is a pleiotropic inflammatory chemokine that has been implicated in various inflammatory disorders, including atherosclerosis [32–34]. Platelet factor 4 promotes vascular inflammation by recruiting monocytes to adhere to damaged endothelial cells in atherosclerosis. Our results indicated that fruitflow decreased PF4 levels in ADP- and collagen-activated platelets. This suggests that fruitflow may inhibit the release of α-granules by platelets and reduce the levels of inflammatory mediators from platelets under pathological conditions.

Previous studies reported that Akt/GSK3β and p38 MAPK is involved in platelet spreading [35, 36]. Syk and PLCγ2 are critical signaling molecules in platelet activation mediated by collagen receptor GPVI [37]. Our results for the first time showed that fruitflow completely prevented platelet spreading and suppressed Akt/GSK3β, p38 MAPK, Syk and PLCγ2 phosphorylation in collagen-stimulated platelets. These results were supported by our proteomic research which reported water-soluble tomato extract fruitflow altering the phosphoproteomic profile of collagen-stimulated platelets [38].

## Conclusions

The present study provides novel evidence for the mechanism of action of fruitflow on inhibiting platelet function. Fruitflow inhibits platelet aggregation and reduces TXB_2_, 6-keto-PGF_1α_, and PF4 levels. The mechanism is related to the inhibition of Akt/GSK3β, Syk/PLCγ2 and p38 MAPK phosphorylation. Fruitflow is a natural product derived from tomato and can be used as a health food for decreasing platelet activity.

## Supplementary Information

**Additional file 1.**

## Data Availability

The datasets that were used and/or analyzed in the present study are available from the corresponding author upon reasonable request.

## Abbreviations

FF: Fruitflow
TXB_2_: Thromboxane B_2_
6-keto-PGF_1α_: 6-keto-prostaglandin F_1α_
PF4: Platelet factor 4
ADP: Adenosine diphosphate
PRP: Platelet-rich plasma
