# Supplementary figures and images for: Fruitflow inhibits platelet function by suppressing Akt/GSK3β, Syk/PLCγ2 and p38 MAPK phosphorylation in collagen-stimulated platelets
Source: BMC Complement Med Ther. 2022 Mar 17;22:75. doi: 10.1186/s12906-022-03558-5 (PMC8932123; doi:10.1186/s12906-022-03558-5)

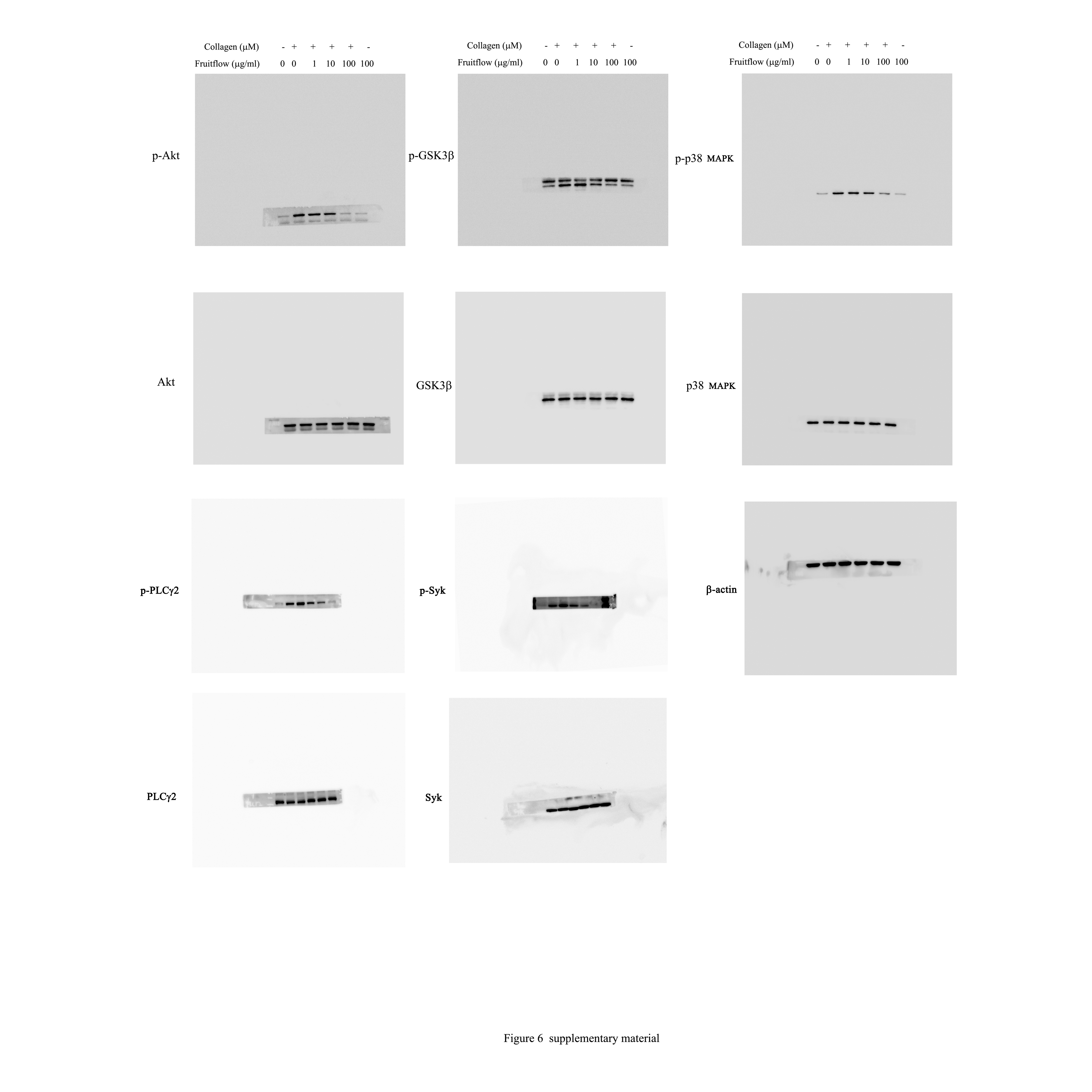

Supplement: Supplementary file 1 — Additional file 1. [file 12906_2022_3558_MOESM1_ESM.tif]
